# Longitudinal changes in functional capacity: effects of socio-economic position among ageing adults

**DOI:** 10.1186/1475-9276-11-78

**Published:** 2012-12-15

**Authors:** Tommi Sulander, Heikki Heinonen, Tuuli Pajunen, Antti Karisto, Pertti Pohjolainen, Mikael Fogelholm

**Affiliations:** 1 Department of Social Research, University of Helsinki, P.O.Box 18, FI-00014, Helsinki, Finland; 2 Age Institute, Asemapäällikönkatu 7, FI-00520, Helsinki, Finland; 3 Department for Lifestyle and Health, National Institute for Health and Welfare, P.O.Box 30, FI-00271, Helsinki, Finland; 4 Max Planck Institute for Demographic Research, Laboratory of Statistical Demography, Konrad-Zuse-Straße, 118057, Rostock, Germany; 5 Department of Food and Environmental Sciences, University of Helsinki, P.O.Box 62, 00014, Helsinki, Finland

**Keywords:** Functional capacity, Socio-economic position, Ageing, Longitudinal

## Abstract

**Introduction:**

Health and functional capacity have improved especially in Western countries over the past few decades. Nevertheless, the positive secular trend has not been able to decrease an uneven distribution of health. The main aim of this study was to follow-up changes in functional capacity among the same people in six years time and to detect whether the possible changes vary according to socio-economic position (SEP). In addition, it is of interest whether health behaviours have an effect on these possible changes.

**Methods:**

This longitudinal follow-up study consisted of 1,898 individuals from three birth cohorts (1926–1930, 1936–40, 1946–50) who took part in clinical check-ups and answered to a survey questionnaire in 2002 and 2008. A sub-scale of physical functioning from the RAND-36 was used to measure functional capacity. Education and adequacy of income were used as indicators of socio-economic position. Repeated-measures ANOVA was used as a main method of analysis.

**Results:**

Physical functioning in 2002 and 2008 was poorest among those men and women belonging to the oldest cohort. Functional capacity deteriorated in six years among men in the oldest cohort and among women in all three cohorts. Socio-economic disparities in functional capacity among ageing people existed. Especially lower adequacy of income was most consistently associated with poorer functional capacity. However, changes in functional capacity by socio-economic position remained the same or even narrowed independent of health behaviours.

**Conclusion:**

Socio-economic disparities in physical functioning are mainly incorporated in the level of functioning at the baseline. No widening socioeconomic disparities in functional capacity exist. Partly these disparities even seem to narrow with ageing.

## Introduction

Health and functional capacity have improved especially in Western countries over the past few decades. Nevertheless, the positive secular trend has not been able to decrease an uneven distribution of health. Cross-sectional and longitudinal studies examining disparities and changes in functional capacity among middle and old age people by socio-economic position (SEP), have shown those with a lower position to have more disabilities [1-8].

Longitudinal changes in functional capacity by SEP have not been studied extensively. Previous evidence suggests that negative longitudinal change of functional capacity among those non-disabled 75-year-old men and women who have poor material wealth is greater compared to those with good material wealth [1]. Adequacy of income as an indicator of SEP has shown to be a strong predictor of disabilities [5]. A longitudinal study from the UK suggested that respondents with lower socio-economic status had a higher number of new incidences of disability, and severity of disability increased more in comparison to those with higher socio-economic status during the follow-up [9]. Similar results have been reported also from the USA [8]. A recent study among 40–50 year old Danish people indicated that onset of mobility disability among disability-free people at baseline was more common in the lower social classes [10].

Some evidence suggests health behaviors to act as mediators from low social position to poor functional capacity among older adults [11]. It has been found out that lower SEP together with lifestyle factors are strongly and independently associated with increased incident locomotor disability [2]. When examining changes in functional capacity it is therefore essential to take into account modifiable forms of health behavior, e.g., physical activity and nutrition together with smoking and alcohol consumption. Maintaining healthy habits not only prevents the deterioration of functional capacity [11-14] but also cognitive capacity and the development of dementia [15,16].

The main aim of this study was to examine longitudinal changes in functional capacity among the same people in three different age cohorts in six years time and to detect whether the possible changes vary according to socio-economic position. It was also of interest whether health behaviours had an effect on these possible longitudinal changes in functional capacity.

## Methods

The analyses are based on the Good Aging in the Lahti Region (GOAL) program: a longitudinal study where three birth cohorts have been followed during ten years (2002–12). The subjects were born in 1926–1930, 1936–1940, and 1946–1950 and were 72–76, 62–66, and 52–56 years old, respectively, at the baseline. The baseline study including a survey questionnaire and clinical check-ups was conducted in 2002 with the target sample of 4,272 persons, and the participation rate was 66% (n = 2,815). As an end point, we used data from the second follow-up survey together with clinical check-ups. It was carried out in 2008, with 1,898 persons of those participating also in 2002. The design and the sample of the study have been described in more detail elsewhere [17].

### Measures

The socio-economic variables included education and adequacy of income. Self-reports of basic education were coded into three categories: elementary school, middle school and secondary school. Adequacy of income was selected as an indicator of socio-economic position as self-rated economic condition is strongly related to health among ageing persons [18]. Although, self-rated economic condition is related to income, it is independent of it, as there may be considerable variation in the severity of financial strains experienced by people having similar incomes [19]. Therefore self-rated economic condition may differentiate people better than income in contrast to health. In fact self-rated economic condition has been found to be a better predictor of health than income [20,21]. We used adequacy of income as an indicator of self-rated economic condition. Adequacy of income after necessary expenses (e.g. cost of living and repayment of a loan) was divided into two categories: very and rather good in the first category and average, rather poor and very poor in the second.

A sub-scale of physical functioning from the RAND-36 [22] was used to measure functional capacity. It is a ten-question scale that captures the following abilities to deal with the physical requirements of life: 1) lifting and carrying a bag of groceries, 2) climbing stairs (one floor), 3) climbing stairs (several floors), 4) activities requiring moderate effort (e.g. hoovering, moving furnitures, brisk walking), 5) activities requiring vigorous effort (lifting heavy objects, jogging), 6) walking about 100 meters, 7) walking about 500 meters, 8) walking about 1000 meters, 9) bending, kneeling, or stooping, 10) bathing or dressing oneself. These ten abilities (with three classes: no limitation = 1, a slight limitation = 2, a lot of limitation = 3) were used to produce a continuous scale ranging from 10 to 30 points. Reliability for this scale according to Cronbach’s alpha was 0.992.

Health behaviour was measured with self-reports of physical activity, use of fruit and vegetables, smoking and alcohol consumption. All these behaviours correlated with each other, and all the other expect alcohol consumption correlated with baseline functional capacity (data not shown).

Physical activity was measured with a question: “How often do you move or strain yourself during your spare time? The scale varied between “Few times a year or less” (1) and “daily” (7). The most common answer among men in different age cohorts was 2–3 times per week (1946–1950: 30%, 1936–1940: 29% and 1926–1930: 27%). Corresponding figures among women were 32%, 29% and 26%.

Based on a question: “How often have you used following foods during the past week (7 days)”, the use of fresh, cooked vegetables and fruits was measured with three questions in four classes (never = 1, during 1 to 2 days = 2, during 3–5 days = 3,during 6–7 days = 4). These three questions were summed up to produce a ten-point scale ranging from 3 to 12 points. The prevalence of those men who scored 9 or more points in different age cohorts was: 1946–1950: 42%, 1936–1940: 43% and 1926–1930: 52%. Corresponding figures among women were: 67%, 69% and 62%.

The indicator of smoking status included current daily smokers (=1) and those not smoking (= 2) (inc. ex- smokers). Male smokers in different age cohorts were: 1946–1950: 26%, 1936–1940: 14% and 1926–1930: 5%. Corresponding figures among females were: 18%, 7% and 1%. The alcohol use scale (how often do you use alcohol?) varied from never =1 to four times or more per week=5. Highest prevalence observed among men was the category of using alcohol 2–4 times a month (1946–1950: 40%, 1936–1940: 34% and 1926–1930: 35%). Correspondingly among women in two youngest cohorts it was monthly or less frequently (1946–1950: 37%, 1936–1940: 39%) and in oldest cohort those who reported not consuming alcohol at all (45%).

### Statistical methods

The IBM SPSS version 19.0 was used to perform the following analyses: (1) the distributions of socio-economic variables (%) by gender and birth cohort (n); (2) means, paired sample *t*-test and the one way analysis of variance for functional capacity by gender and birth cohort; and (3) repeated-measures ANOVA [23] was used to detect any univariate differences in functional capacity by categories of socio-economic variables by gender and birth cohort. Two models examining time-by-group interactions were constructed. First model shows unadjusted results for each socio-economic variable. Second model shows same results adjusted by health behaviours.

## Results

Physical functioning both in 2002 and 2008 was strongly related to age cohort being poorest among those men and women belonging to the oldest cohort (1926–1930) and best among the youngest age cohort (1946–1950). Functional capacity deteriorated in six years among men in the oldest cohort and among women in all three cohorts (Table 1).

**Table 1:** Functional capacity (mean and SD) in the study cohorts by sex

		**Cohort**						**Oneway**
		**1946-1950**		**1936-1940**		**1926-1930**		**ANOVA**
**Functional capacity**		**Mean**	**sd**	**Mean**	**sd**	**Mean**	**sd**	
Men	2002	12,4	3,4	13,7	4,0	15,4	4,8	1<2<3
	2008	12,7	3,7	13,9	4,3	16,4***	4,8	1<2<3
Women	2002	12,7	3,7	14,6	4,5	17,2	5,0	1<2<3
	2008	13,2**	3,8	15,3***	4,6	18,9***	4,9	1<2<3

Longitudinal change in functional capacity by time and cohort.

** p < 0.01, *** p < 0.001.

Men in cohorts 1926–1930 and 1936–1940 were less educated than men in the youngest cohort (1946–1950) (Table 2, percentages in parenthesis). A significant time-by-group interaction concerning education was observed for functional capacity among youngest male birth cohort (Table 2). Those with secondary school education had better baseline functioning than those who had background of elementary school, but they experienced a greater decline in functional capacity during 6 years. This result remained after adjusting for health behaviours. Men with fair or poor adequacy of income had worse functional capacity than those with good adequacy of income both at baseline and at follow-up even when adjusting for health behaviours (Table 2). However, there was no time-by-group interaction concerning adequacy of income.

**Table 2:** Results of repeated-measures ANOVA among men

**MEN**						**Model 1**		**Model 2**	
		**Baseline**		**6-year follow-up**		**Results of**		**Results of**	
**Cohort 1946–1950 (n = 278)**		Mean	SE	Mean	SE	ANOVA		ANOVA	
Education	Elementary school (69%)	12,6	3,5	12,6	3,5	*Within Subjects*		*Within Subjects*	
	Middle school (18%)	12,4	3,5	13,0	4,0	Phys. Capacity	**p=0.012**	Phys. Capacity	**p=0.003**
	Secondary school (13%)	11,5	2,2	13,2	4,3	*Between Subjects*		*Between Subjects*	
						Education	p=0.888	Education	p=0.771
Adequacy of income	Good (62%)	11,7	2,6	12,2	3,2	*Within Subjects*		*Within Subjects*	
	Fair or poor (38%)	13,5	4,2	13,5	4,3	Phys. Capacity	p=0.214	Phys. Capacity	p=0.292
						*Between Subjects*		*Between Subjects*	
						Adeq. Of income	**p<0.001**	Adeq. Of income	**p<0.001**
**Cohort 1936–1940 (n = 350)**									
Education	Elementary school (80%)	13,8	4,1	14,1	4,4	*Within Subjects*		*Within Subjects*	
	Middle school (13%)	13,8	4,0	13,5	3,5	Phys. Capacity	p=0.497	Phys. Capacity	p=0.788
	Secondary school (7%)	11,9	2,6	12,5	4,1	*Between Subjects*		*Between Subjects*	
						Education	p=0.075	Education	p=0.375
Adequacy of income	Good (64%)	13,1	3,5	13,5	4,0	*Within Subjects*		*Within Subjects*	
	Fair or poor (36%)	14,8	4,7	14,7	4,6	Phys. Capacity	p=0.190	Phys. Capacity	p=0.658
						*Between Subjects*		*Between Subjects*	
						Adeq. Of income	**p=0.001**	Adeq. Of income	**p=0.004**
**Cohort 1926–1930 (n = 221)**									
Education	Elementary school (81%)	15,7	4,8	16,8	4,8	*Within Subjects*		*Within Subjects*	
	Middle school (9%)	13,8	4,1	14,6	4,9	Phys. Capacity	p=0.662	Phys. Capacity	p=0.372
	Secondary school (10%)	14,8	4,9	15,1	4,5	*Between Subjects*		*Between Subjects*	
						Education	p=0.080	Education	p=0.129
Adequacy of income	Good (58%)	15,0	4,6	15,9	4,8	*Within Subjects*		*Within Subjects*	
	Fair or poor (42%)	16,0	5,0	17,1	4,9	Phys. Capacity	p=0.762	Phys. Capacity	p=0.279
						*Between Subjects*		*Between Subjects*	
						Adeq. Of income	p=0.074	Adeq. Of income	p=0.283

Model 1: Sociodemographic measure.

Model 2: Sociodemographic measure+ smoking status, physical activity, consumption of fruits and vegetables, alcohol consumption.

In the birth cohort 1936–40 at the baseline, better adequacy of income was associated with better functional capacity at both study points among men (Tables 2). However, there were no clear disparities in changes in functional capacity. Level of functional capacity and changes in functioning by educational groups were similar. Those men in the oldest cohort who had poorer SEP according to education and adequacy of income had slightly poorer functional capacity. However, results were not statistically significant (Table 2). Changes in functioning among men in the oldest cohort followed similar pattern in all subgroups.

Women in the oldest cohort were less educated and had lower prevalence of good adequacy of income than those women in the youngest cohort (Table 3, percentages in parenthesis). Women with fair or poor adequacy of income had worse functional capacity than those with good adequacy of income when adjusting for health behaviours (Table 3). As among men, there was no time-by-group interaction concerning adequacy of income among women. In the cohort born in 1936–40, better adequacy of income was associated with better functional capacity at both study points among women. In the oldest cohort, women with better adequacy of income had better baseline functioning than those with a poorer adequacy of income, but they experienced slightly greater decline in functional capacity during 6 years (Table 3). No disparities in changes in functional capacity by education among women could be seen, especially when adjusting for health behaviours.

**Table 3:** Results of repeated-measures ANOVA among women

**WOMEN**						**Model 1**		**Model 2**	
		**Baseline**		**6-year follow-up**		**Results of**		**Results of**	
**Cohort 1946–1950 (n = 374)**		**Mean**	**SE**	**Mean**	**SE**	**ANOVA**		**ANOVA**	
Education	Elementary school (58%)	12,9	3,9	13,4	4,0	*Within Subjects*		*Within Subjects*	
	Middle school (27%)	12,5	3,3	13,0	3,6	Phys. Capacity	p=0.992	Phys. Capacity	p=0.983
	Secondary school (15%)	12,2	3,6	12,7	3,4	*Between Subjects*		*Between Subjects*	
						Education	p=0.243	Education	p=0.242
Adequacy of income	Good (70%)	12,2	3,4	12,9	3,7	*Within Subjects*		*Within Subjects*	
	Fair or poor (30%)	13,8	4,1	13,9	3,9	Phys. Capacity	p=0.104	Phys. Capacity	p=0.124
						*Between Subjects*		*Between Subjects*	
						Adeq. Of income	**p=0.001**	Adeq. Of income	**p=0.002**
**Cohort 1936–1940 (n = 405)**									
Education	Elementary school (73%)	14,9	4,5	15,4	4,6	*Within Subjects*		*Within Subjects*	
	Middle school (18%)	13,9	4,1	14,8	4,6	Phys. Capacity	p=0.148	Phys. Capacity	p=0.220
	Secondary school (9%)	13,4	4,1	14,8	4,7	*Between Subjects*		*Between Subjects*	
						Education	p=0.251	Education	p=0.627
Adequacy of income	Good (60%)	14,0	4,2	14,7	4,5	*Within Subjects*		*Within Subjects*	
	Fair or poor (40%)	15,5	4,8	16,1	4,8	Phys. Capacity	p=0.956	Phys. Capacity	p=0.474
						*Between Subjects*		*Between Subjects*	
						Adeq. Of income	**p=0.001**	Adeq. Of income	**p=0.008**
**Cohort 1926–1930 (n = 236)**									
Education	Elementary school (76%)	18,0	5,1	19,6	4,9	*Within Subjects*		*Within Subjects*	
	Middle school (17%)	14,9	3,5	17,0	4,2	Phys. Capacity	p=0.676	Phys. Capacity	p=0.220
	Secondary school (7%)	14,5	4,5	16,8	4,8	*Between Subjects*		*Between Subjects*	
						Education	**p<0.001**	Education	p=0.627
									
Adequacy of income	Good (57%)	15,6	4,1	17,7	4,4	*Within Subjects*		*Within Subjects*	
	Fair or poor (43%)	19,4	5,3	20,4	5,0	Phys. Capacity	**p=0.033**	Phys. Capacity	**p<0.001**
						*Between Subjects*		*Between Subjects*	
						Adeq. Of income	**p<0.001**	Adeq. Of income	**p<0.001**

Model 1: Sociodemographic measure.

Model 2: Sociodemographic measure+ smoking status, physical activity, consumption of fruits and vegetables, alcohol consumption.

## Discussion

Functional capacity deteriorated in six years among men in oldest cohort and among women in all three cohorts. This study showed SEP disparities, especially disparities by adequacy of income in functional capacity. Poorer adequacy of income was most consistently associated with poorer functional capacity. However, changes in functional capacity over time by SEP remained in most parts the same or even narrowed independent of health behaviours.

Our results indicate steady or partly decreasing SEP disparities in functional capacity. This is partly in accordance with earlier results. In a study from the USA [8] it was found out that poorer women had worse functioning at baseline independent of health-related covariates. However, poverty status was unrelated to lower extremity function decline over three years. Rautio et al. [24] found out that higher income and better education were related to better physical capacity among 75-year old people living in Jyväskylä, Finland, but decline in physical capacity in five and ten year periods was parallel in all socio-economic groups. The association between income and physical capacity remained after adjusting for health behaviour. Similar educational disparities in disability have also been found in other studies [3]. In the present study no clear disparities by education was found. As the number of participants in different age cohorts were rather small, it is possible that there was not enough statistical power to reveal educational disparities.

Our results are in accordance with a study from the UK [5], where it was found out in longitudinal analyses that self-perceived adequacy of income was a strong predictor of onset of disability. Adequacy of income has not been extensively studied in relation to functional capacity. However, previous studies that have used it in analyses have consistently shown it to be a strong predictor of health and functional capacity [5,18,20,21] Even though adequacy of income as an indicator of SEP has not been comprehensively rationalized in previous literature, it is an indicator which is partly independent of income. Therefore there may be considerable variation in the severity of the financial strains experienced by people having similar incomes [19]. This might be one explanation why increasing number of studies use it when examining socioeconomic disparities in health.

As different studies have various designs, there exist contradictory results. For instance a Danish study concerning 40–50 year old people found out that those with lower social class had a higher onset of mobility disability [10]. Similar results have been suggested from China [25]. In the Danish study, however, people were disability-free at baseline. In the present study it was of interest to examine changes in average scores of functional capacity in different age cohorts by SEP, not only those who are disability-free at baseline.

Results from the present study indicate that adjusting for health behaviours does not have an effect on disparities or changes in functional capacity by SEP. This is in accordance with some previous evidence [24]. It has been concluded previously that health behaviour has a strong impact on functional capacity [2,26]. Correlation analyses revealed (data not shown) that all the behaviours used in the present study correlated statistically significantly with each other and all the health behaviours excpet alcohol consumption correlated significantly with baseline functional capacity. Even though alcohol consumption was not individually associated with functional capacity, there is previous evidence that health behaviours are interrelated with each other [27]. Especially smoking has shown to have strongest and most consistent associations with other unhealthy behaviours [28]. These interactions between various behaviours might have an impact on the level of functional capacity. It seems that according to population groups examined in this study, health behaviours and their possible interactions may result in the accumulation of advantages or disadvantages in a longer time span.

There are a few points that may explain why health behavior in this study did not had an impact on functional capacity. First a relatively short follow-up time might explain why health behaviour did not turn out to be a stronger predictor of later functional capacity. Second, the health behaviour between various SEP’s did not differ so that it would have had an impact on later functioning. The health behaviour measures used in this study were based on large Finnish nationwide follow-up surveys which have been carried out since 1970s. However, it is possible that the measures were not sensitive enough to reveal the potential impact of health behaviour on later functioning.

This study had several strengths. The study design was longitudinal which enabled the investigation of causal relationships. The data included information on two strong measures of socio-economic position, which made it possible to assess the individual effects of different aspects of social determinants. Another strength was that we had data on health-related covariates which may act as mediators from socio-economic position to ill health and poor functional capacity.

However, our data had some limitations as well. The data was collected from three cohorts born 10 years apart from each other, which made it complicated to combine the data-sets thus giving somewhat small sample sizes for multivariate methods. Also, the follow-up time was relatively short. Especially in the younger cohorts, changes in functional capacity probably occur during a longer period.

The sub-scale of physical functioning produced from the RAND-36 does not allow a comparison of various sum-scores with the quality point of view. Thus it is difficult to interpret what kind of exact differences in difficulties people have if they have for instance scores of 12 or 14 points. Anyhow, high Chronbach’s alpha score of the scale used in the present study indicated very good internal consistency of the scale. Furthermore, many functional capacity scales have a clear hierarchy between the various abilities. It is shown, for instance, that over 90% of older people who have difficulty in dressing and undressing have also difficulty in mobility [11]. In this sense, we can assume that people scoring higher in the present study have somewhat poorer functional capacity.

## Conclusion

Socio-economic position in the present study had an effect on functional capacity but not to its decline. The physical functioning of the ageing people deteriorated during a six-year follow-up, especially among women. A longer follow-up period, however, might be needed to detect marked differences in functional decline. Also, it seems that socio-economic differences in functioning were already incorporated in the level of functioning at the baseline. A positive message is that according to this study, health inequality concerning functional capacity has not widened but it has remained steady or even narrowed.

## Competing interests

The authors declare that they have no competing interests.

## Authors’ contributions

TS, HH, TP, AK, PP and MF designed the study. TS and TP did the literature review and wrote the first draft. HH and TS analysed the data and implemented the statistical analysis. HH, TP, AK, PP and MF made in-depth revisions to the first draft and HH made further guidance to the statistical analysis. TS made the final version according to the comments from HH, TP, AK, PP, and MF. TS, HH, TP, AK, and MF had full access to the data, and TS had final responsibility for the submission. All authors read and approved the final manuscript.
